# Circadian rhythms, Neuroinflammation and Oxidative Stress in the Story of Parkinson’s Disease

**DOI:** 10.3390/cells9020314

**Published:** 2020-01-28

**Authors:** Alexandre Vallée, Yves Lecarpentier, Rémy Guillevin, Jean-Noël Vallée

**Affiliations:** 1 DACTIM-MIS, Laboratory of Mathematics and Applications (LMA), UMR CNRS 7348, University of Poitiers, CHU de Poitiers, 86021 Poitiers, France; remy.guillevin@chu-poitiers.fr; 2 Centre de Recherche Clinique, Grand Hôpital de l’Est Francilien (GHEF), 77100 Meaux, France; yves.c.lecarpentier@gmail.com; 3 CHU Amiens Picardie, University of Picardie Jules Verne (UPJV), 80000 Amiens, France; valleejn@gmail.com; 4 Laboratory of Mathematics and Applications (LMA), UMR CNRS 7348, University of Poitiers, 86021 Poitiers, France

**Keywords:** circadian rhythms, Parkinson’s disease, oxidative stress, neuroinflammation

## Abstract

Parkinson’s disease (PD) is one of the main neurodegenerative disease characterized by a progressive degeneration of neurons constituted by dopamine in the substantia nigra pars compacta. The etiologies of PD remain unclear. Aging is the main risk factor for PD. Aging could dysregulate molecular pathways controlling cell homeostatic mechanisms. PD cells are the sites of several metabolic abnormalities including neuroinflammation and oxidative stress. Metabolic structures are driven by circadian rhythms. Biologic rhythms are complex systems interacting with the environment and controlling several physiological pathways. Recent findings have shown that the dysregulation of the circadian rhythms is correlated with PD and its metabolic dysregulations. This review is focused on the key role of circadian rhythms and their impact on neuroinflammation and oxidative stress in Parkinson’s disease.

## 1. Introduction

Parkinson’s disease (PD) is one of the main neurodegenerative diseases (ND) characterized by a progressive degeneration of neurons constituted by dopamine in the substantia nigra pars compacta. PD is triggered in the brainstem or in the spinal cord of patients who stay asymptomatic for a long time [1,2]. The etiologies of PD remain unclear but the presence of Lewy bodies (clumps of alpha-synuclein and ubiquitin proteins in neurons which are detectable in post-mortem brain histology) has been shown. PD is marked by tremor symptom, rigidity, bradykinesia, and postural instability. These symptoms occur only when the main part of dopaminergic (DAergic) cells is lost in the substantia nigra pars compacta, which means that the smooth, coordinated regulation of striatal motor circuits is also lost [3]. Depression or rapid eye movement (REM)-associated sleep behavior disorder (RBD) are non-motor symptoms that could precede the onset of pathology.

Aging is the main risk factor for NDs. Aging could dysregulate molecular pathways controlling cell homeostatic mechanisms. Neurodegenerative cells are the sites of several metabolic abnormalities [4]. Numerous metabolic mechanisms can induce and initiate neurodegenerative mechanisms. PD shows a metabolic remodeling entailing increased oxidative stress and neuroinflammation [5,6]. Metabolic structures are driven by circadian rhythms (CRs) [7,8,9,10]. CRs are directly implicated in the regulation of the metabolic pathways observed in neurodegenerative disease and especially in PD [11,12]. Metabolic dysregulation observed in PD is the consequence of an energy metabolism reprogramming induced by CRs. We focused this review on the key role of CRs and their impact on neuroinflammation and oxidative stress in Parkinson’s disease.

## 2. Circadian rhythms (CRs)

CRs are major biological phenomena found in all universal processes. Their endogenous characteristic is an innate oscillation associated with a period about over one day. All the studied organisms show this oscillatory process. Numerous cell functions present temporal variations driven by these oscillatory and circadian rhythms, including gene expression, metabolic reprogramming, and molecular and cellular pathways. Different integration levels allow the study of CRs as endocrinal, physiological, and neuronal cell behaviors. Although the coordination of CRs is organized by specific pacemaker structures, primary circadian oscillations are controlled at the cell level. Oscillations are determined by numerous clock genes [13]. The control of the circadian clock is based on an intracellular temporal tracking system allowing organisms to change direction and thus adapt their behavior and physiology of their life span [14]. In animals, it is well known that a specific set of transcription factors constitutes the molecular architecture of the circadian clock. These determinants are used in both positive and negative feedback which are modulated in a cell-autonomous manner [15].

Endogenous oscillations generate a freewheeling period which is close to 24 h to maintain for the organism constant ambient conditions. Circadian oscillations are the product of the post-transcriptional modifications of proteins [16]. A cell-autonomous transcriptional auto-regulatory complex feedback loop operates with clock gene transcriptional activators and in turn clock genes with a negative feedback role inhibit their expression by disrupting the activity of their activators. Clock and Bmal1 core clock genes encode activators and *Period* (Per) and *Cryptochrome* (Cry) encode repressors [17]. The input pathways of the retinohypothalamic tract senses external timing signals of environmental information (dark/light for example) which interact with the different compounds of the oscillators (i.e., the circadian clock genes). The oscillators are synchronized with the 24 h solar day. This time of day generated by the input pathways is transposed by the oscillators to output pathways to control and then to regulate the expression of circadian clock genes and thus the rhythmicity. Moreover, output pathways are predicted to be rhythmic and controlled by transcription factors or signaling. The factors activated by the circadian clock, in turn, regulate downstream the circadian clock genes in a time-of-day-specific manner [18]. This system can synchronize with its environmental time by its internal clock. To respect the environment, the input pathways are vital to maintain this timing for oscillators. At the process level, named entrainment, the input pathways can reset the activity of the oscillators to stay in the 24 h period of the environment [18]. Environmental cues can be detected by input pathways which in turn can modulate many mechanisms to control the activity or level of compounds of oscillators to keep a correct time of day expression. This phenomenon is observed in several environmental cues, including nutrition, social interactions, and temperature [19,20]. Furthermore, the clock allows a strategy named “gating” to restrict responses to environmental cues at some times of day. Diurnal mammals are insensitive to a light pulse during the day. However, during the night, a light pulse can advance or delay the clock to synchronize diurnal mammals with the environment [15]. Environmental signals can interact with molecular oscillators in some cells in complex multicellular organisms. In unicellular organisms, the cell is modulated by oscillators in response to light [21]. However, in multicellular organisms, not all cell types have sensory capabilities (as photoreception) leading to clock oscillation. The oscillators, and thus the overall rhythmicity of organisms are concentrated into compounds including a master pacemaker and peripheral oscillators [22]. Faced with these sensory inputs, rather than individual cells, the organism presents some nervous systems which possess environmental cue abilities as central oscillators or pacemakers. In humans, sensory clock inputs are localized in the brain, where signals from the master pacemaker lead to oscillators in some tissues of the organism.

Nonvisual retinal ganglion cells receive and perceive light and transmit this information to the master pacemaker (localized in the hypothalamus) through neural connections. The central pacemaker synchronizes oscillators to other tissues by using circadian input pathways from the nervous system to peripheral cell systems. Moreover, to maintain the entrainment of these peripheral oscillators by the environment, this central system ensures that cellular oscillations within tissues are properly in phase to provide resonance between individual cellular rhythms [23]. Melatonin operates as a synchronizer in humans and provides temporal feedback to oscillators within the nervous system to control the circadian phase and the stability of the rhythm [24]. In humans, as in other mammals, melatonin is considered as the main influencer of CRs through its action on receptors in the nervous system [25].

## 3. Circadian Clock

**Figure 1:**
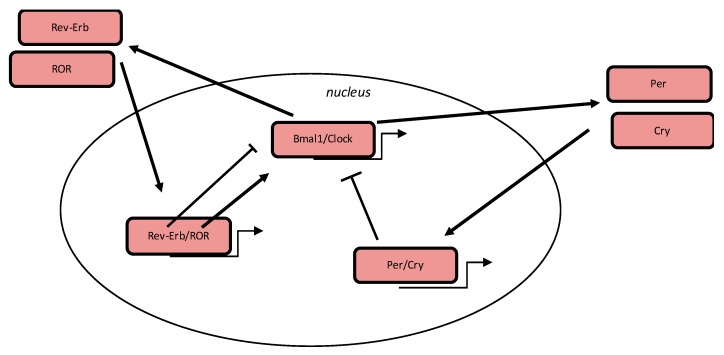
Circadian clock genes. The clock process is a stimulatory circle, involving the Bmal1/Clock heterodimer which activates the transcription of *Period* (Per) and *Cryptochrome* (Cry) genes, and the inhibitory feedback circle with the Per/Cry heterodimer which translocates to the nucleus and which represses the transcription of the Clock and Bmal1 genes. An additional circle implicates the retinoid-related orphan receptors (RORs) and Rev-Erb factors with a positive feedback by RORs and a negative feedback by Rev-Erb.

Some biological mechanisms in humans (such as metabolic pathways, lipid and glycose homeostasis, and autophagy) are controlled by the circadian “clock” (circadian locomotor output cycle kaput) (Figure 1). The circadian clock is present in the hypothalamic suprachiasmatic nucleus (SCN). CRs are endogenous and have entrainable free-running 24 h periods. Numerous transcription factors can act on the CRs. These factors are called circadian locomotor output cycle kaput (*Clock*), brain and muscle aryl-hydrocarbon receptor nuclear translocator-like 1 (*Bmal1*), Period 1 (*Per1*), Period 2 (*Per2*), Period 3 (*Per3*), and Cryptochrome (*Cry 1 and Cry 2*) [26,27]. These factors are controlled by positive and negative self-loop-regulation modulated by CRs [15,28]. *Clock* and *Bmal1* heterodimerize leading to the transcription of *Per1, Per2, Cry1 and Cry2* [29]. The *Per/Cry* heterodimer downregulates its stimulation through a negative feedback. This heterodimer translocates back to the nucleus to directly inhibit the *Clock/Bmal1* complex and then to downregulate its transcription [29]. The *Clock/Bmal1* complex stimulates the transcription of retinoic acid-related orphan nuclear receptors, *Rev-Erb* and retinoid-related orphan receptors (*RORs*). Through a positive feedback loop, *RORs* activate the transcription of *Bmal1*, whereas through a negative feedback loop, *Rev-Erb* downregulates its transcription [29].

## 4. CRs in PD

**Figure 2:**
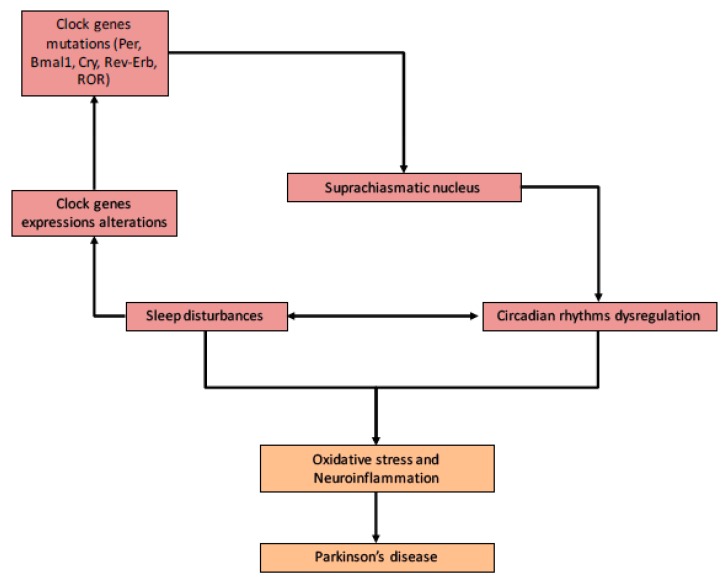
Circadian rhythms (CRs) and Parkinson’s disease (PD). Relationship between PD, CRs, oxidative stress, and neuroinflammation. Alterations in clock genes and the melatonin pathway contribute to the dysregulation of circadian sleep rhythmicity. CRs deregulation leads to metabolism alterations (i.e., oxidative stress) and neuroinflammation contributing to PD.

Many studies have observed that the core clock mechanism is present in neurons and astrocytes [30,31]. Circadian dysregulation can occur in aging pathogenesis, cancers, and chronic diseases [32,33,34]. Aging is characterized by some modifications in the circadian system [12]. Aging is marked by changes in circadian rhythmicity, reducing amplitude, increasing intra-daily variability, and decreasing inter-daily stability of CRs (Figure 2) [35,36,37].

Many studies have highlighted an association between CRs and PD (Table 1) [38,39,40]. PD patients present low levels of peak activity and amplitude of the rest–activity cycle [12]. The observed increase of physical activity and the reduction of immobility periods during the night leads to activity of the diurnal motor without oscillations [12].

In parallel, dopamine can regulate the rhythmicity of *Per2* expression [41,42]. Striatal dopamine controls the *Bmal1/Clock* heterodimer expression [43] in a receptor-dependent manner [44]. TH (tyrosine hydroxylase), the enzyme responsible for the synthesis of dopamine, and the dopaminergic receptors show daily fluctuations [45].

CRs increase the PD neuropathology [46] although PD presents a day-to-day progression in the deterioration of the motor function [47,48]. A circadian fluctuation in PD can explain the deregulation in motor and visual performance [49]. Altered blood pressure and heart rate are common in PD. High blood pressure and heart rate have been observed under the light phase and low blood pressure has been shown during the dark phase [50]. During the day, the sympathetic activity of PD patients decreases in association with a loss of circadian rhythmicity of heart rate variability and with the lower melatonin sympathetic morning peak [51]. In PD, the elevation of cortisol and the diminution of melatonin are correlated with the alteration of the *Bmal1* expression [52]. *Cry1* and *Per1* levels are diminished in the PD rotenone model [53], while melatonin administration can restore the level of *Per1* but not *Cry1* and *Bmal1* levels [53]. CR systems can modulate the hypothalamic–pituitary–adrenal (HPA) axis and can restore it by promoting the dopamine function [54]. CRs are regulated by dopamine at the behavioral levels [55]. Decreased levels of *Per2* in dorsal striatum of rats have been observed with the diminution of dopamine by 6-hydroxydopamine (6-OHDA) [41]. The activation of D2 receptors in the dopamine-depleted striatum leads to the restoration of *Per2* activity [56]. During the dark span of PD, *Bmal1* expression is decreased and its expression is associated with the severity of the disease [57]. In PD, alteration of the Bmal1 levels are correlated with dopamine diminution [43,52]. Dopamine diminution can affect the central compound of the molecular clock and circadian disruption, which leads to the acceleration of PD progression [58].

## 5. Oxidative stress in PD

**Figure 3:**
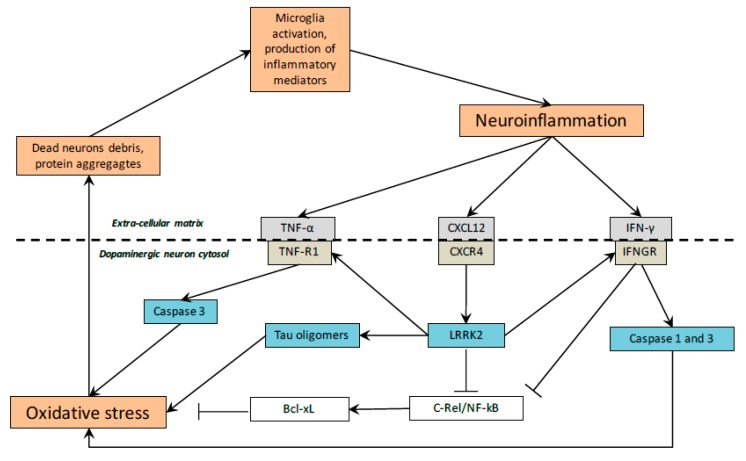
Neuroinflammation and oxidative stress in PD. Neuronal death induced by neuroinflammation is caused by several pathways involving oxidative stress in PD. Bcl-xL: B-cell lymphoma-extra-large; CXCL12: C-X-C motif chemokine ligand 12; IFN-γ: interferon-γ; LRRK2: leucine-rich repeat kinase 2; NF-κB: nuclear factor κB; TNF: tumor necrosis factor.

Many studies have shown the increase of oxidative stress in PD [80]. Mitochondrial dysregulation have been observed in PD through the increased production and release of reactive oxygen species (ROS) [81]. Mitochondrial depletion leads to cell damage and death by the diminution of energy production through the enhancement of oxidative stress [82]. Oxidative stress and mitochondrial dysregulation lead to cell death and dementia [83,84,85]. The initiation of PD is correlated with oxidative stress enhancement [5]. Inhibition of the respiratory chain activity in substantia nigra pars compacta of PD is associated with the increase of ROS production and the induction of apoptosis [81,86,87] (Figure 3).

The human body generates free radicals of oxygen for oxidative metabolism. During aerobic respiration, molecular oxygen (O_2_) is reduced to water molecules in each mitochondrion. By this mechanism, O_2_, H_2_O_2,_ and OH are produced by a leakage of oxygen [6]. Phagocytic cells, during infections, can generate high levels of NO, O_2,_ and H_2_O_2_ to defend the body and thus to reduce infection. But, these produced radicals can also destroy the body cells [88].

Some enzymes, including monoamine oxidase (MAO), L-amino acid oxidase, and tyrosine hydroxylase, are involved in dopamine metabolism and in the production of ROS [89]. In parallel, inflammation is considered another source of ROS production. Nevertheless, many pathways can confluence with ROS. In microglia, the aggregation of ROS-induced proteins can induce inflammation [90]. Four mechanisms involved in PD are correlated with inflammation and ROS: the increase of iron levels, the decrease of glutathione (GSH) levels, the reduction of 26S proteasomal activity, and the impairment of mitochondrial complex I regulation [91,92]. In the physiological step, MAO produces H_2_O_2_. In PD, H_2_O_2_ is transformed in hydroxyl radicals (OH) by iron via the Fenton’s reactions. Thus, H_2_O_2_ and OH produce ROS [93]. GSH is oxidized by H_2_O_2_ and OH in the cytoplasm [94] leading to GSH leakage in PD. The leakage of GSH molecules leads to the conversion of glutamate and cysteine into glutamyl peptides and cysteinyl peptides. These peptides are toxic for dopaminergic cells by crossing the cellular membrane and by activating ROS into dopaminergic neurons. These peptides downregulate the activity of complex I of the mitochondria leading to ROS production and enhancement of oxidative stress [95]. Dopaminergic (DAergic) cells cannot repair misfolded proteins in PD due to the impairment of the proteasome [96]. Oxidative stress enhances the carbonylation of proteins, which is an irreversible and unrepairable modification. Carbonylation is a phenotype of cellular senescence leading to the aggregation of proteins. Protein aggregation is one of the major pathological features of nigrostriatal DAergic neurons in PD. These aggregated proteins induce neuroinflammation and oxidative stress [97].

## 6. CRs and Oxidative Stress

Several studies in both animals and plants have shown that the production of ROS and anti-oxidants is temporally regulated by CRs [98,99]. The diminution of the expression of *Bmal1* is associated with the mitochondrial dysfunction leading to the increase of ROS in organs [100]. Moreover, mitochondrial dysregulation and oxidative stress have been involved in age-related diseases [101].

In Drosophila, dysregulation of *Per* implicates circadian oscillations in the deregulation of oxidative stress marker levels [102]. *Per* inhibition enhances oxidative injury and shortens lifespan [103,104]. Flies with carbonyl reductase mutation present *Per* deletion, accelerating neurodegeneration and causing neural oxidative injuries [103]. Oxidative damages in the cortex and neurodegeneration are correlated with *Bmal1* depletion [105]. In the brain, Bmal1 can control the transcription of numerous redox defense genes, such as *Nqo1* and *Aldh2* [105]. *Nqo1* encodes NADPH dehydrogenase, a critical redox defense enzyme and Aldh2 activates aldehydes during mitochondrial respiration to prevent oxidative damages and degeneration [106].

## 7. Inflammation in PD

Inflammation is one of the major cause of PD. Research studies have shown that inflammation plays a major role in PD (Table 1, Figure 3) [59]. Inflammation leads to activation of the apoptosis pathways in dopaminergic cell during PD development [107,108]. The correlation between inflammation and PD is mutual; inflammation leads to death of dopaminergic cells but DAergic cell death can stimulate, in a vicious feedback, inflammation [109]. Additionally, the relationship between Parkinson’s disease and inflammation is mutual; while inflammation leads to dopaminergic cells death, DAergic cell death can develop or augment inflammation, that is inflammation is both cause and effect in the story of DAergic cell death [109]. Moreover, inflammatory factors induce oxidative stress, which force DAergic cells to activate death signals [110]. Some inflammatory activators have a main role in PD [111]. The stimulation of microglia leads to the activation of their pro-inflammatory enzymes (such as inducible nitric oxide synthase and cyclooxygenase) and the release of pro-inflammatory cytokines (such as C-X-C motif chemokine ligand 12 (CXCL12), tumor necrosis factor-α (TNF-α), interferon-γ (IFN-γ), interleukin (IL)-6, and IL-1β [112]. The NF-kB pathway has a major role in the production of these proinflammatory enzymes and cytokines in microglia [113]. TNF-α leads to apoptosis by TNF-R1 receptor death domain activating the caspases 1 and 3 [60]. TNF-α also downregulates c-Rel–NF-kB. c-Rel–NF-kB presents a neuroprotective role through the inhibition of apoptosis via B-cell lymphoma-extra-large pathway in dopaminergic neurons [113]. High-levels of CXCR4 (called fusin) expression and its ligand CXCL12 have been shown in PD. The complex formed by CXCR4–CXCL12 activates caspase 3, which leads to neural cell death via apoptosis [61,114]. The complex of IFN-γ–IFNGR signaling phosphorylates leucine-rich repeat kinase 2 (LRRK2) protein [62]. LRRK2 interacts with numerous cell processes in microglia and dopaminergic neurons. Activated LRRK2 protein inhibits the expression of c-Rel–NF-kB. Thus, inflammation is increased by the insufficiency of c-Rel–NF-kB [63,64]. LRRK2 activation is responsible for the formation of tau oligomers, which activate cell death signals [115,116]. LRRK2 controls some vesicles trafficking in cells and its overexpression is associated with the augmentation of inflammatory cytokines [117].

## 8. CRs and Inflammation 

Some diurnal variations have been shown in Ly6Chi monocytes, which are the main line of defense against infection (Figure 2, Table 1) [65]. Bmal1 can act as an anti-inflammatory factor by inhibiting CCL2 expression. CCL2 is a chemokine which stimulates monocyte recruitment to the infection site by binding to the E-box present on the *Ccl2* gene promoter region. Moreover, *Bmal1* possesses two microRNA-binding sites in its 3′-UTR leading to the dysregulation of clock control and CRs control of inflammation [118]. Deficiency in Bmal1 is associated with high levels of IL-6 in response of lipopolysaccharide stimulation [66].

The *Clock* gene can acetylate the receptor of glucocorticoid receptors leading to the dysregulation of the immune system [67]. The overstimulation of *Clock* expression is correlated with phosphorylation and acetylation of p65 leading to the enhancement of transcriptional activity of the NF-κB pathway [68]. Some studies have shown that the dysregulation of the *Clock* activity is associated with infection [119].

*REV-ErBα* can inhibit *IL-6* expression [66]. In macrophage cells, Rev-Erbα can decrease *Ccl2* expression by directly binding to the RORE motif on the promoter region of *Ccl2* gene [69]. Moreover, *Bmal1* and *REV-Erbα* can cooperate in the regulation of *Ccl2* expression [70]. Bmal1 is responsible for the regulation of *RORα* which can induce the transcription of IκBα, an inhibitor of NF-κB pathway [71]. Deficiency in *Per2* is associated with diurnal variations in IFN-gamma expression leading to the impairment function of natural killer cells [120]. *Per2* knockout reduces levels of TNF-α and IL-12 [121]. In macrophages, *Per2* mRNA rhythms act as inverse phase to Bmal1 [122]. *Per2* is considered as a negative compound of the CR feedback loop and then, can promote inflammation through the inhibition of the complex *Bmal1/Clock* [123]. Moreover, *Per2* can also decrease *REV-ErBα* function responsible for activation of inflammation response [123]. Deficiency in *Cry1* and *Cry2* leads to CAMP overproduction and PKA activity (protein kinase A) increasing p65 phosphorylation and NF-κB pathway [124]. Administration of anti-TNF-α is associated with both reduction of inflammation and Cry expression [72].

## 9. Melatonin: A Potential Therapeutic Drug in PD

Melatonin (also named 5-methoxy-N-acetyltryptamine) is a natural product of the pineal gland [125]. Melatonin controls the regulation of sleep (Table 1) [126,127]. Its production has been shown during darkness and thus, may participate in sleep circadian regulation [128,129]. Diminution of melatonin amplitude rhythms is correlated with aging [37,130], and its deregulation has been shown in numerous neurodegenerative diseases (such as Alzheimer’s disease and Parkinson’s disease) [131]. Melatonin presents some effects, such as anti-inflammatory, anti-oxidant, and neuroprotective effects [128,132,133,134,135,136]. Melatonin increases the potential of mitochondrial membrane, activates mitochondrial biogenesis [137], and increases the mitochondrial function [138].

## 10. Melatonin and Oxidative Stress in PD

In PD, few studies have investigated the different effects of melatonin on oxidative stress. Melatonin administration is associated with the increase of superoxide dismutase (SOD), GSH, and mitochondrial complex-I activity in the rat model of PD. In parallel, melatonin administration is correlated with the decrease of OH and the increase of catalase [73]. GSH expression, and GSSG/GSH ratio are increased after melatonin administration in the rat model of PD [74]. Malondialdehyde (MDA) expression, a marker of oxidative stress, is decreased while SOD is increased under melatonin administration in rat model of PD induced by 6-OHDA or by MPTP [75]. In the same way, other models of PD rats induced by rotenone have shown the increase of both GSH and SOD after melatonin administration [76]. In PD, melatonin can decrease the number of degenerating neurons and lipid peroxidation [139]. Recent studies have shown that melatonin is associated with the decrease of oxidative stress in rats of PD induced by MPP(+) [140] and in mice of PD induced by MPTP [141]. Few studies in humans have investigated the role of melatonin on oxidative stress in PD. In PC12 cells, melatonin administration is associated with the protection against the reduction of mRNAs of antioxidant enzymes evoked by 6-OHDA [77], with the reduction of apoptosis and necrosis [78], and with the prevention of mitochondrial dysregulation [79].

## 11. Melatonin and Neuroinflammation in PD

Few studies have focused on the effects of melatonin on inflammation in PD. Melatonin administration can prevent the increase of iNOS in PD mice-induced MPTP [142]. iNOS, increased in striatum and substantia nigra of PD, leads to stimulation of nitric oxide production and neuroinflammation in PD [143]. COX2 is responsible for the involvement of neuroinflammation in PD [143]. COX2 expression is decreased under melatonin administration in PD mice-induced MPTP [141].

## 12. Conclusions

Aging is involved in the pathophysiology of PD and dysregulates several molecular pathways controlling cell homeostasis. Metabolic abnormalities have been observed in PD. This review highlights the main role of both inflammation and oxidative stress in PD. These signals operate together to alter the cellular activity in PD. In parallel, CRs can control metabolic structures and thus, can regulate the metabolic pathways involved in PD. A metabolic reprogramming expressed by oxidative stress in concordance with inflammation operates in PD under the control of CRs. Looking for signs of early circadian dysregulation could help to diagnose PD before the onset of known symptoms or could allow the rapid introduction of therapeutic strategies. Melatonin, a major marker of CRs, could be an interesting target in PD therapeutic strategy. However, few studies have focused on the interactions between melatonin with oxidative stress and inflammation in PD. Future clinical trials could be implemented to study the efficacy of melatonin in PD by directly targeting these two signals.

## Figures and Tables

**Table 1:** CRs, oxidative stress, inflammation and Melatonin in PD.

	Factors	Targets	Models	References
CRs in PD	Per2	D2 dopaminergic receptor	Rat dorsal striatum	[42]
	Clock/Bmal1	D2 dopaminergic receptor	D2R knockout mice	[43]
	Per, Bmal1	D2 receptor agonist quinpirole	Mouse striatum	[44]
	Diurnal motor variation	Levodopa therapy	PD patients	[48]
	Heart rate variability	Diurnal and frequency low	PD patients	[51]
	Bmal1, Per, Rev-Erb	Augmentation of sleep latency, diminution of sleep efficacy and diminution of rapid eye movement sleep	PD patients	[52]
	Per, Cry, Bmal1	Diminution of serotonin metabolism, diminution of melatonin	RIPD male wistar rat	[53]
	Bmal1	DA system, 6-OHDA blunt period	Dorsal striatum rat	[56]
	Bmal1	Pittsburgh sleep quality index score	PD patients	[57]
Inflammation in PD	HLA-DR-positive reactive microglia	Diminution of cortical choline acetyltransferase activity	PD patients	[59]
	TNF alpha	Augmentation of caspase 1 and 3	PD patients	[60]
	CXCR4	Augmentation of microglia activity	Post-mortem PD patients	[61]
	LRRK2	IFN gamma response	PD patients	[62]
	LRRK2	IL-1, Cox2, NF-kappaB augmentation	Microglia cells	[63]
	LRRK2	TNF alpha, NF-kappaB augmentation	PD patients	[64]
CRs and inflammation in PD	Bmal1	Ly6C(hi) inflammatory monocyte	Monocytes	[65]
	Rev-Erb	IL-6	Patient inflammatory diseases	[66]
	Clock	Histone acetyltransferase	Clock-out cells	[67]
	Clock/Bmal1	NF-kappaB	Mouse model	[68]
	Rev-Erb	Ccl2 expression	C57BL/6J mice	[69]
	Rev-Erb	TH17	Nfil3(−/−) mice	[70]
	ROR	IL-1beta, IL-6	Wild type and staggerer (RORalpha(sg/sg)) mice	[71]
	Cry	TNF-alpha, IL-1beta, IL-6	Cry1(−/−)Cry2(−/−) mice	[72]
Melatonin in PD	Melatonin	Mitochondrial complex 1 activity	Hcy rat model of PD	[73]
	Melatonin	GSH levels	Rat model of PD	[74]
	Melatonin	6-OHDA levels	Hemiparkinsonian rat model	[75]
	Melatonin	GSH levels, SOD levels	Hemiparkinsonian rat model	[76]
	Melatonin	mRNAs of antioxidants	PC12 cells	[77]
	Melatonin	Apoptosis, necrosis	PC12 cells	[78]
	Melatonin	Caspase 3/7	PC12 cells	[79]

## Abbreviations

Bmal1: brain and muscle aryl-hydrocarbon receptor nuclear translocator-like 1; Clock: circadian locomotor output cycles kaput; PD: Parkinson’s disease; Per: period; RORs: retinoid-related orphan receptors. Bcl-xL: B-cell lymphoma-extra-large; CXCL12: C-X-C motif chemokine ligand 12; IFN-γ: interferon-γ; LRRK2: leucine-rich repeat kinase 2; NF-κB: nuclear factor κB; TNF: tumor necrosis factor.
